# Novel *PTPRQ* variants associated with hearing loss in a Chinese family *PTPRQ* variants in Chinese hearing loss

**DOI:** 10.3389/fgene.2024.1399760

**Published:** 2024-08-14

**Authors:** Yuan Hou, Yuanzhen Shi, Longyan Liu, Shihong Duan

**Affiliations:** Department of Otolaryngology-Head and Neck Surgery, Lanzhou University Second Hospital, Lanzhou, China

**Keywords:** protein tyrosine phosphatase receptor Q, Non-syndromic hearing loss, hereditary disease, *PTPRQ*, next-generation sequencing

## Abstract

**Introduction:**

Hearing loss is one of the most prevalent congenital sensory disorders. Over 50% of congenital hearing loss cases are attributed to genetic factors. The *PTPRQ* gene encodes the protein tyrosine phosphatase receptor Q, which plays an important role in maintaining the structure and function of the stereocilia of hair cells. Variants in the *PTPRQ* gene have been implicated in hereditary sensorineural hearing loss.

**Methods and Results:**

Utilizing next-generation sequencing technology, we identified novel compound heterozygous variants (c.977G>A:p.W326X and c.6742C>T:p.Q2248X) in the *PTPRQ* gene within a Chinese national lineage, marking the first association of these variants with hereditary sensorineural hearing loss.

**Discussion:**

Our findings further emphasize the critical role of *PTPRQ* in auditory function and contribute to a more comprehensive understanding of *PTPRQ*-associated hearing loss mechanisms, aiding in clinical management and genetic counseling.

## 1 Introduction

Hearing loss is a prevalent congenital sensory disorder. The World Health Organization (WHO) estimates that by 2050, approximately 2.5 billion individuals globally will experience hearing loss, with at least 700 million enduring moderate or more severe forms (World Health Organization, 2021). Notably, hereditary factors are anticipated to account for nearly half of these cases (World Health Organization, 2021). Non-syndromic hearing loss (NSHL) constitutes approximately 70% of congenital genetic hearing impairments (Sheffield and Smith, 2019). In the majority of NSHL cases (80%), the hearing loss correlates with biallelic pathogenic variants and is typically inherited in an autosomal recessive pattern (Shearer et al., 1993).

To date, 153 genes implicated in NSHL have been identified, including 63 genes associated with autosomal dominant non-syndromic hearing loss (ADNSHL) and 86 genes associated with autosomal recessive non-syndromic hearing loss (ARNSHL) (Hereditary Hearing Loss, retrieved 28 May 2024, from https://hereditaryhearingloss.org/).

*PTPRQ* is located on chromosome 12q21.31, comprising 58 exons (Schraders et al., 2010). It encodes protein tyrosine phosphatase receptor Q, which plays an important role in maintaining the stereocilia structure and function of hair cells (Goodyear et al., 2003). Its loss or dysfunction may result in shaft connector malformation of stereocilia in the inner ear (Goodyear et al., 2003). The loss of *PTPRQ* may cause the detachment of the hair cell’s apical membrane from the underlying actin cytoskeleton, allowing the membranes of adjacent stereocilia to “zipper” upward (Goodyear et al., 2012). This process prevents the fusion of stereocilia and results in both structural and functional impairments in vestibular hair cells, predominantly observed within the first 2 weeks of life in mutant mice (Goodyear et al., 2012). By 3 months of age, these impairments manifest as either undetectable vestibular evoked potentials or significantly elevated thresholds (Goodyear et al., 2012). Variants in the *PTPRQ* gene cause hereditary sensorineural hearing loss. The Online Mendelian Inheritance in Man (OMIM) database (https://www.omim.org/entry/603317, accessed on 28 May 2024) indicates that DFNB84A (MIM 613391), a variant of ARNSHL, is associated with *PTPRQ* variants, typically resulting in progressive moderate to profound familial hearing loss (Shahin et al., 2010). Additionally, some *PTPRQ* variants may lead to ADNSHL, such as DFNA73(MIM 617663) (Eisenberger et al., 2018). Through whole-exome sequencing (WES), we identified two novel compound heterozygous variants (c.977G>A and c.6742C>T) in the *PTPRQ* gene within a Chinese family. These variants have not been previously reported in association with ARNSHL. Our findings enhance the understanding of genotype-phenotype correlations in *PTPRQ*-related hearing loss and may inform clinical management and genetic counseling.

## 2 Materials and methods

### 2.1 Family description

**FIGURE 1:**
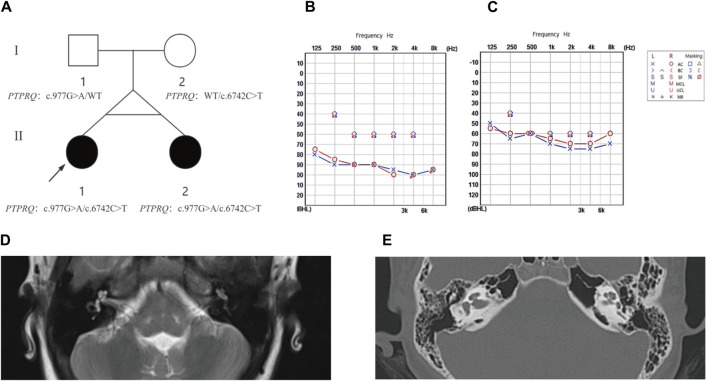
The family pedigree, audiometric, and imaging results for proband II-1 and sibling II-2. **(A)** Pedigree analysis: This figure depicts the family pedigree of monozygotic twins proband II-1 and sibling II-2, who exhibit delayed progressive SNHL. Notably, their parents had no history of hearing loss. Both twins possess a compound heterozygous variant in the *PTPRQ* gene, comprising alleles c.977G>A and c.6742C>T. The father carries the c.977G>A heterozygous variant, and the mother harbors the c.6742C>T heterozygous variant. Affected individuals are highlighted in black, while WT indicates the wild type. **(B)** Audiogram for proband II-1: This audiogram reveals severe to profound bilateral hearing loss. **(C)** Audiogram for sibling II-2: This audiogram indicates moderate to severe bilateral hearing loss. **(D)** MRI Findings for Proband II-1 shows normal anatomical structures of the middle and inner ear. **(E)** HRCT Findings for Sibling II-2 confirms normal morphology of the middle and inner ear structures.

We studied a 13-year-old girl (II-1) and her twin sister (II-2), both of whom had delayed progressive post-linguistic NSHL (Figure 1). They belong to a non-consanguineous, Han Chinese family from Jinchang City, Gansu Province, China, with no known history of hearing loss. A detailed clinical history was obtained for all participants, and phenotypes were assessed via otoscopy, physical examination, and pure tone audiometry (PTA); auditory and speech performance were also evaluated. Apart from deafness, no additional clinical symptoms were observed. The study underwent review and approval by the Medical Ethics Committee of the Second Hospital of Lanzhou University. Written informed consent was obtained from all subjects in compliance with the Ethics Committee of the Second Hospital of Lanzhou University. For the minors involved in the study, parental consent was provided.

### 2.2 Clinical examination

The proband (II-1) and related family members underwent otoscopy in the Department of Otolaryngology-Head and Neck Surgery, Lanzhou University Second Hospital, and the degree of their hearing loss was assessed via PTA. Magnetic resonance imaging (MRI) of the temporal bone for II-1 and high-resolution computed tomography (HRCT) for II-2 were performed to evaluate the integrity of middle and inner ear structures, including the cochlea and vestibule. All clinical assessments, including physical examinations, otoscopy, and medical histories, were documented at the outpatient clinic of the Second Hospital of Lanzhou University.

### 2.3 Sample collection

Blood samples were collected from the proband and related family members in the Department of Otolaryngology-Head and Neck Surgery, Lanzhou University Second Hospital. Following the provision of written consent, 4–6 mL of peripheral venous blood were collected using vacuum blood collection tubes with EDTA anticoagulant. Subsequently, the samples were stored at a temperature of −80°C and transported under conditions that maintained a low temperature.

### 2.4 Variant detection and analysis

In order to ascertain the genetic etiology of HL in the proband and her sister, we conducted WES on DNA samples obtained from the blood of the two individuals. Subsequently, validation testing was conducted using DNA extracted from the blood of their parents.

WES was conducted at Genesky Biotechnologies Inc., Shanghai (201315) using the SureSelectXT Reagent kit for library preparation and the SureSelectXT Human All Exon V6 kit for probe hybridization, aimed at all-exon capture. Subsequently, target regions were captured using the Agilent V6r2 all-exon capture chip, and sequencing was performed on the Illumina NovaSeq 6000 platform. The methodology is outlined below:

The quality of the sample genomic DNA was evaluated using two methods. Agarose gel electrophoresis was employed to assess the integrity of the genomic DNA, requiring that the electrophoretic bands be distinctly visible without significant smearing. Additionally, a Nanodrop 2000 instrument was used to determine the concentration and quality of the genomic DNA, with the requirements that the concentration exceed 50 ng/μL, the total sample amount be at least 1.5 μg, and the OD260/280 ratio fall within the range of 1.8–2.0.

The raw data derived from sequencing underwent quality control analysis with FastQC, resulting in clean reads after stringent filtering. These reads were then aligned to the human reference genome (GRCh38/hg38) using the Burrows-Wheeler Aligner (BWA). The alignment results for each sample were analyzed using Picard software. This analysis included counting and proportioning sequences, assessing the percentage of duplicate reads from PCR amplification in sample preparation and exon capture experiments, and evaluating the ratios of Q20 and Q30 quality sequences, average coverage depth, and the coverage range from 1 × to 100 × in exon regions (Table 1). The preliminary alignment results obtained from BWA were corrected using the GATK standard procedure, which targeted duplicate sequences due to PCR amplification, misalignments caused by indels, and base quality issues. After correction, the output files were used to analyze sequencing coverage and depth for target regions, single nucleotide variants (SNVs), and indel calls. The GATK Haplotype Caller method was used to detect SNVs/Indels in each sample, with filtering performed according to the software’s recommended guidelines. All SNVs/Indels were compared against the latest population, functional, and disease databases using ANNOVAR to assess their variant frequency, functional features, conservation, and pathogenicity. Variant pathogenicity was predicted using tools such as BayesDel addAF, ClinPred, CADD, DANN, and Inter Var, following ACMG guidelines for variant pathogenicity assessment. Primers designed from gene variant sites identified through WES in probands were used in Sanger sequencing to verify gene variant sites in other family members, analyzing the co-segregation of genotype and phenotype, tracing the variant’s origin within the family, and providing evidence for genetic analysis.

**TABLE 1:** Sample Genome Alignment Results. TOTAL_READS: Total number of reads utilized in genome alignment. PF_READS: Number of reads that passed quality filters. PCT_PF_UQ_READS_ALIGNED: Percentage of reads uniquely aligned. PCT_USABLE_BASES_ON_BAIT: Percentage of bases correctly aligned to the target region. MEAN_BAIT_COVERAGE: Average coverage depth across bait regions. 1X∼100X: Proportion of exon regions covered at depth levels from 10 × to 100 ×, as determined by comparative sequence analysis. Q20 and Q30: Mapping quality score. PCT_EXC_DUPE: Percentage of sequences excluded due to PCR duplication during library construction.

Sample	TOTAL_READS	PF_READS	PCT_PF_UQ_READS_ALIGNED	PCT_USABLE_BASES_ON_BAIT	MEAN_BAIT_COVERAGE	10X	20X	30X	40X	50X	100X	Q20	Q30	PCT_EXC_DUPE
II-1	52519392	52519392	0.996245	0.561515	72.678578	0.981135	0.90665	0.771807	0.625227	0.496933	0.157749	0.978230694	0.940470833	0.33287
II-2	39386338	39386338	0.994236	0.529482	51.386372	0.964226	0.822109	0.61686	0.437688	0.307253	0.059905	0.974153846	0.932154566	0.33714

## 3 Result

### 3.1 Clinical result

The proband (II-1) developed hearing loss at age eight, which progressively worsened over the subsequent year. Her twin sister (II-2) exhibited identical clinical symptoms without any developmental delays or regression. Utilizing PTA (Figure 1), we confirmed that the proband exhibited severe to profound NSHL, while individual II-2 experienced moderate to severe NSHL. Neither showed additional symptoms of inner ear damage, such as vertigo or dizziness, and imaging techniques, such as CT of the temporal bone and MRI of the brain, showed no anatomical anomalies. Comprehensive otoscopic and physical examinations, focusing on renal, ophthalmological, and electrocardiographic aspects, detected no systemic abnormalities (Figure 1). Notably, the mother (I-2) had no history of miscarriage or stillbirth, and the parents, not consanguineously married, reported no familial hearing loss, were in good health, and had no significant medical or traumatic histories. The mother experienced an uneventful pregnancy, with no medications or infections, and the probands were born via a full-term cesarean section, exhibiting normal health post-delivery.

### 3.2 Variant identification data

**FIGURE 2:**
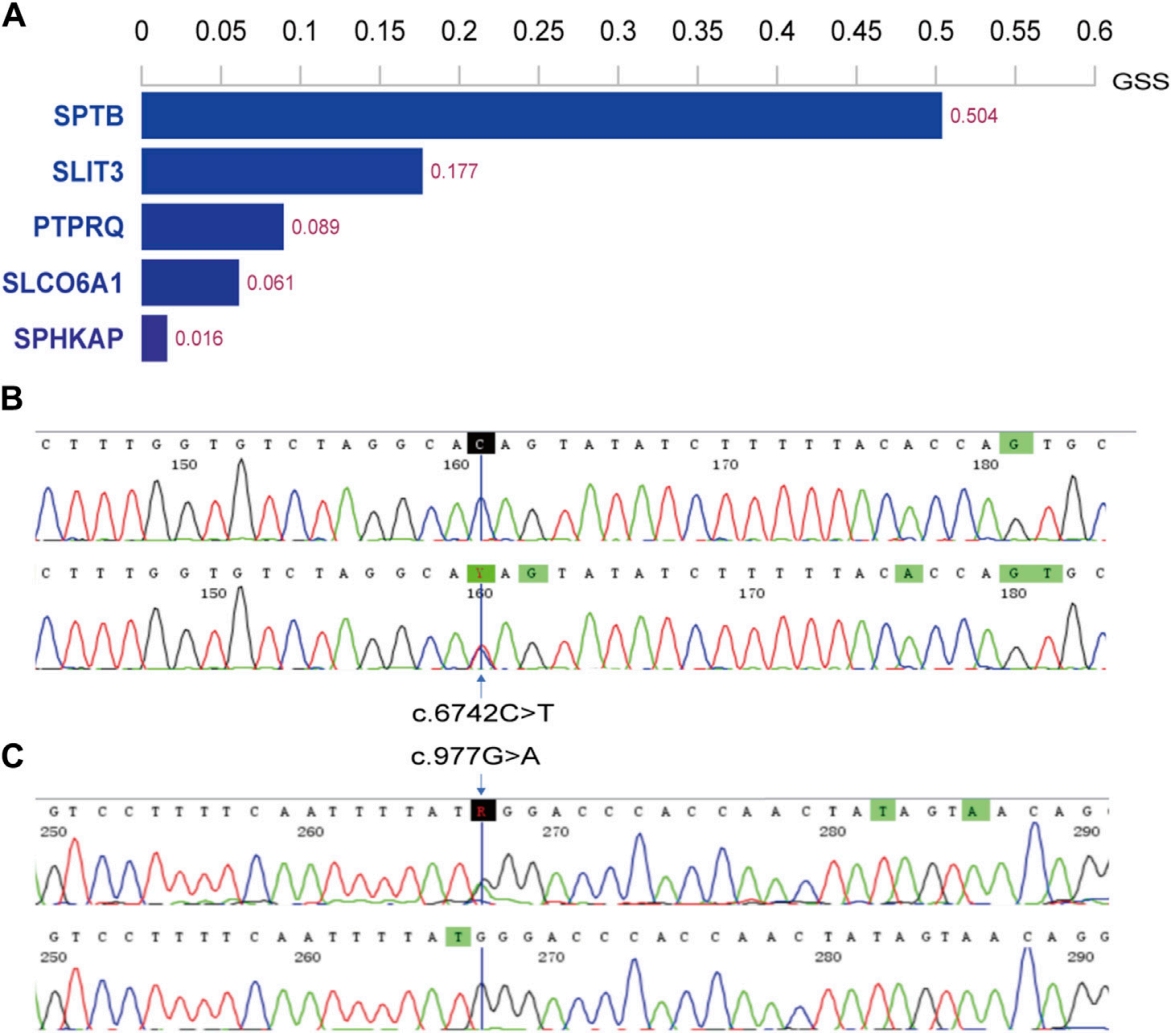
Histogram of top 5 scoring genes and Sanger sequencing results in family members. **(A)** Histogram displaying the top 5 scoring genes based on their Genetically Standardized Score (GSS), with a scoring interval from 0 to 1; **(B)** the mother (I-2) of the proband harbors the c.6742C>T variant; **(C)** the father (I-1) carries the c.977G>A variant.

The WES results were aligned with the human reference genome (GRCh38/hg38). We identified variants in the patients’ genomes, including single nucleotide variants (SNVs) and small insertions/deletions (InDels), from these alignments. For all SNV/InDel loci, we applied the following criteria: loci were retained for further analysis if they met frequency thresholds in several databases—1000 Genomes (less than 0.01), ExAC0.3 Asian population (less than 0.01), and gnomAD Asian population (less than 0.01). Additionally, we considered only those loci that were exonic or splice site mutations and were nonsynonymous. These variants were annotated and assessed for potential pathogenicity, considering their location, type, presence in conserved regions, and known associations with pathogenicity. Given the recessive inheritance pattern observed in this family, the top five genes were identified (Figure 2), with the *PTPRQ* gene showing a strong association with deafness.

Two potential causative loci, c.977G>A:p.W326X and c.6742C>T:p.Q2248X, were identified, both characterized by premature translational termination and localized to exons (Table 2). The samples displayed heterozygosity at these loci. Sanger sequencing confirmed that these variants co-segregate with the disease within the family, as evidenced by heterozygosity in both parents (Figure 2). Specifically, the father carries the c.977G>A variant and the mother the c.6742C>T variant (Figure 1). The two daughters, identified as the probands, demonstrate compound heterozygosity for these variants. These variants were not recorded in the dbSNP, 1000 Genomes, or EXAC databases.

**TABLE 2:** The two variants detected by probands.

Gene	Nucleotide variation	Amino acids Variation	variants type	Exon	Pure/heterozygous	Frequency in normal population	Clinical significance	Origins
*PTPRQ*	c.977G>A	p.W326X	stopgain	7	HET	—	pathogenic	II-1 II-2
*PTPRQ*	c.6742C>T	p.Q2248X	stopgain	44	HET	—	pathogenic	II-1 II-2

### 3.3 Functional analysis of the mutant protein

**FIGURE 3:**
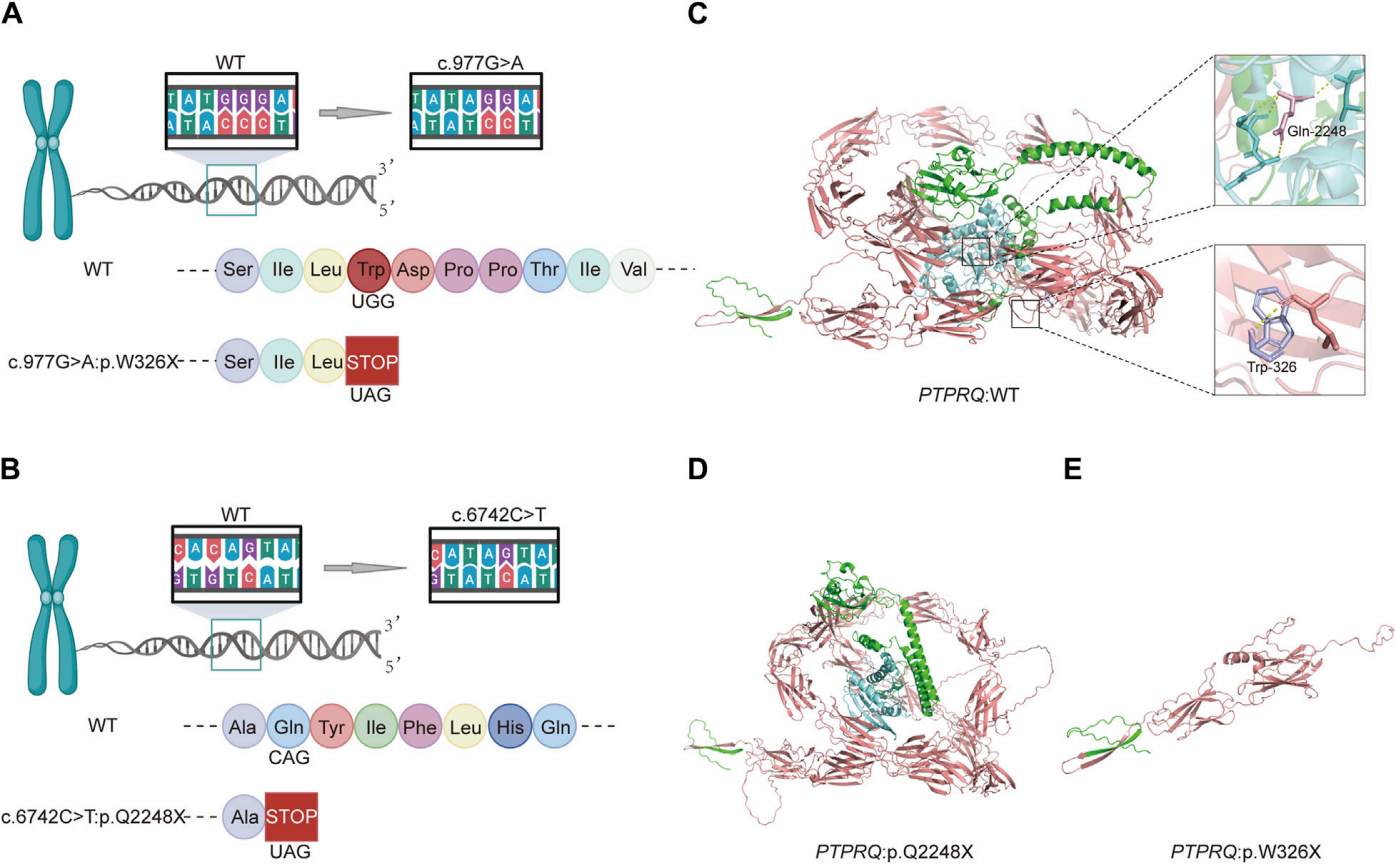
Schematic diagrams of the c.977G>A and c.6742C>T variants and predictive models of the mutant proteins: **(A)** Amino acid coding diagram for the c.977G>A variant; **(B)** Amino acid coding diagram for the c.6742C>T variant; **(C)** WT *PTPRQ* proteins displaying Trp at position 326 in purple and Gln at position 2248 in pink; **(D, E)** Three-dimensional protein modeling reveals that p.W326X and p.Q2248X introduce premature termination codons at amino acids 326 and 2248, respectively, resulting in truncated proteins that impair their normal function.

The variant c.977G>A, located in exon 7, substitutes guanine (G) with adenine (A) at position 977 of the CDS sequence. This substitution changes the TGG codon, which encodes tryptophan (Trp), to the TAG stop codon, terminating amino acid synthesis and resulting in a truncation variant that impairs protein function. Similarly, the variant c.6742C>T, situated in exon 44, replaces cytosine (C) with thymine (T) at position 6742 of the CDS sequence, altering the CAG codon, which encodes glutamate (Glu), to the TAG stop codon. This change also halts amino acid synthesis, leading to a truncation variant that negatively impacts protein functionality (Figure 3). The three-dimensional structures of proteins affected by the two variants were predicted using AlphaFold and PyMOL. It was discovered that the p.W326X and p.Q2248X variants introduce premature termination codons at amino acids 326 and 2248, respectively. These variants lead to the production of truncated proteins that compromise their normal function (Figure 3). In particular, the truncation of p.W326X occurs at the fibronectin (FN) type III domain, which is extracellular, while that of p.Q2248X occurs at the intracellular catalytic site of tyrosine-protein phosphatase (PTPase).

### 3.4 Pathogenicity

Upon searching the databases, we determined that the variants were absent in several key genetic databases: the Human Gene Mutation Database (HGMD), Genome Aggregation Database (gnomAD), Exome Aggregation Consortium (ExAC), ClinVar, and Single Nucleotide Polymorphism Database (dbSNP). Both variants were predicted to be deleterious loci using the software BayesDel addAF, Pred, ClinPred, CADD (https://cadd.gs.washington.edu/), Dann, and InterVar (Table 3). According to the American College of Medical Genetics and Genomics (ACMG) guidelines, both variants were classified as pathogenic (Table 2).

**TABLE 3:** Pathogenicity prediction results for two loci. MutationTasterPred: A = disease_causing_automatic, D = disease_causing, N = polymorphism, P = polymorphism_automatic; BayesDel: T = Tolerated, D = Damaging; ClinPred: T = Tolerated, D = Damaging; CADD Raw: Single Nucleotide Variant (SNV) risk score, with higher values indicating a greater likelihood of being simulated (or “not observed”) and thus more likely to have deleterious effects; the cutoff is typically set at 4; DANN: SNV risk score, with the cutoff usually set at 0.93; VEST: score ranges from 0 to 1, with higher scores indicating a higher likelihood of the mutation causing functional changes, and a score >0.5 is generally considered a potential pathogenic candidate; InterVar: clinical interpretation of variants according to ACMG scoring rules; InterVar_evidence: results based on 28 ACMG scoring criteria; Interpro Domain: protein domain for variants.

Nucleotide variation	MutationTaster Pred	BayesDel addAF Pred	ClinPred	Cadd raw	Dann	VEST score	InterVar	InterVar_evidence	Interpro_domain
c.977G>A	A	D	D	8.428	0.995	0.479	Pathogenic	PVS1 = 1 PS = [0, 0, 0, 0, 0] PM = [0, 1, 0, 0, 0, 0, 0] PP = [0, 0, 1, 1, 0, 0] BA1 = 0 BS = [0, 0, 0, 0, 0] BP = [0, 0, 0, 0, 0, 0, 0, 0]	Fibronectin type III
c.6742C>T	A	D		8.898	0.997	0.467	Pathogenic	PVS1 = 1 PS = [0, 0, 0, 0, 0] PM = [0, 1, 0, 0, 0, 0, 0] PP = [0, 0, 1, 1, 0, 0] BA1 = 0 BS = [0, 0, 0, 0, 0] BP = [0, 0, 0, 0, 0, 0, 0, 0]	Tyrosine-protein phosphatase domain

## 4 Discussion

In this study, patient II-1 exhibited severe to profound hearing loss, while Patient II-2 experienced moderate to severe hearing loss. Both cases were attributed to variants in the *PTPRQ* gene, which is predominantly associated with postlingual deafness and follows an autosomal recessive inheritance pattern (DFNB84A type). Out of the documented cases involving *PTPRQ* variants, approximately seven also reported postlingual onset of symptoms (Table 4).

**TABLE 4:** Pathogenic variants of *PTPRQ* associated with HL. */X: Indicates the formation of an early termination codon; N/A indicates not available.

Origins	Nucleotide variation	Amino acid changes	variants type	Exon	Phenotype	Vestibular dysfunction	reference
Chinese	c.977G>A	p.W326X	Nonsense	Exon 7	Moderate to profound postlingual progressive-delayed SNHL	No	This study
Chinese	c.6742C>T	p.Q2248X	Nonsense	Exon 44	Moderate to profound postlingual progressive-delayed SNHL	No	This study
Japanese	c.279T>G	p.Y93*	Nonsense	Exon 3	Moderate SNHL	No	Sakuma et al. (2024)
Japanese	c.1433_1436del	p.S479Kfs*7	Frameshift (premature stop)	Exon 10	Profound SNHL	N/A	Sakuma et al. (2024)
Japanese	c.4815C>A	p.Y1605*	Nonsense	Exon 28	Profound SNHL	N/A	Sakuma et al. (2024)
Chinese	c.5426 + 1G > A		Splice site	Exon 33	postlingual-delayed progressive SNHL	N/A	Qin et al. (2023)
Chinese	c.90C > A	p.Y30X	Nonsense	Exon 2	postlingual-delayed progressive SNHL	N/A	Qin et al. (2023)
India	c.4006C > T	p.Gln1336Ter	Nonsense	Exon 24	postlingual progressive sensorineural/mixed HL	Yes	Vanniya et al. (2022)
Chinese	c.6603–3 T > G		Splice site	Intron 42	Mild to severe prelingual SNHL	N/A	Jin et al. (2022)
Chinese	c.997 G > A	p.Trp326X	Nonsense	Exon 7	Mild to severe prelingual SNHL	N/A	Jin et al. (2022)
Chinese	c.1057_1057delC	p.L353SfsX8	Frameshift (premature stop)	Exon 8	SNHL	N/A	Yang et al. (2021)
Chinese	c.552delC	p.D184fs	Frameshift		SNHL	N/A	Sang et al. (2019)
French	c.1148G > A	p.Gly383Glu	Missense		Severe age-related HL	N/A	Boucher et al. (2020)
French	c.2521C > T	p.Arg841Trp	Missense		Severe age-related HL	N/A	Boucher et al. (2020)
Chinese	c.4472C > T	p.T1491M	Missense	Exon 26	Severe-profound HL	No	Wu et al. (2018)
Chinese	c.1973T > C	p.V658A	Missense	Exon 13	Severe-profound HL	No	Wu et al. (2018)
Iranian	c.2599T > C	p.Ser867Pro	Missense	Exon 17	Prelingual SNHL	N/A	Talebi et al. (2018)
Germany	c.6881G > A	p.Trp2294*	Nonsense	Exon 45	Mild to severe SNHL	No	Eisenberger et al. (2018)
Algerine	c.5592dup	p.Glu134Glyfs*6	Frameshift (premature stop)	Exon 32	Profound SNHL	N/A	Ammar-Khodja et al. (2015)
Algerine	c.6080dup	p.Asn2027Lys*9	Missense (premature stop)	Exon 38	Profound SNHL	No	Ammar-Khodja et al. (2015)
Chinese	c.16_17insT	p.L8fsX18	Frameshift (premature stop)	Exon 15	Severe to profound prelingual SNHL	N/A	Sang et al. (2015)
Chinese	c.2714delA	p.E909fsX922	Frameshift (premature stop)	Exon 39	Severe to profound prelingual SNHL	N/A	Sang et al. (2015)
Chinese	c.5981A > G	p.E1994G	Missense	Exon 37	Moderate to profound prelingual SNHL	No	Gao et al. (2015)
Chinese	c.3125A > G	p.D1042G	Missense	Exon 20	Moderate to profound prelingual SNHL	No	Gao et al. (2015)
Japanese	c.745C>T	p.R249*	Nonsense	Exon 6	Severe SNHL	N/A	Sakuma et al. (2015)
Japanese	c.1261C > T	p.Arg421X	Nonsense	Exon 9	progressive profound SNHL	Tinnitus, no history of vertigo	Sakuma et al. (2015)
Japanese	c.166C > G	p.Pro56Ala	Missense	Exon 3	non-progressive profound SNHL (c.166C>G/1261C>T)	No	Sakuma et al. (2015)
Japanese	c.6453 + 3delA		Splice site	Exon 41	non-progressive moderate SNHL (c.6453 + 3delA/c.4640T > C)	Tinnitus, no history of vertigo	Sakuma et al. (2015)
Japanese	c.4640T > C	p.Met1349Thr	Missense	Exon 27			Sakuma et al. (2015)
Palestinian	c.1285C > T	p.Gln429X	Nonsense	Exon 9	Moderate to severe prelingual SNHL	N/A	Shahin et al. (2010)
Moroccan	c.1369A > G	p.Arg457Gly	Missense	Exon 19	moderate SNHL with progressive HL in left ear	Yes	Schraders et al. (2010)
Dutch	c.1491T > A	p.Tyr497X	Nonsense	Exon 19	progressive profound SNHL	Yes	Schraders et al. (2010)

In summary, we identified two novel compound heterozygous variants in the *PTPRQ* gene in two sisters experiencing postlingual HL, which is presumed to be inherited in an autosomal recessive manner. Both variants result in premature termination of translation, leading to the production of truncated proteins through nonsense-mediated mRNA decay. This decay affects the functionality or results in the absence of function in the extracellular, transmembrane, and phosphatase structural domains. The proband exhibited progressive hearing loss without vestibular dysfunction symptoms such as tinnitus or vertigo. However, vestibular evoked potentials were absent in most *PTPRQ* knockout mice, and the reason for this difference remains unclear (Goodyear et al., 2012; Sakuma et al., 2015).

A total of 32 pathogenic *PTPRQ* variants associated with hearing loss have been reported (Table 4). We have summarized the corresponding nucleotide and amino acid changes. The majority of these variants were detected in Asian populations, predominantly in Chinese and Japanese groups, and have also been observed in European (Germany, France and the Netherlands) and African (Algeria and Morocco) countries. The two variants identified in this study are novel and previously unreported.

*PTPRQ* encodes a membrane protein of the stereocilia, which plays a key role in the function of the hair cell as a sound receptor in the acoustic system. Protein tyrosine phosphatase receptor-type Q consists of three domains: an extracellular domain with 18 FNIII repeats, a short hydrophobic transmembrane region, and an intracellular domain featuring a single consensus PTPase catalytic site (Wright et al., 1998). It belongs to the type III tyrosine phosphatase receptor (R3 PTPR) family (Zhang et al., 2022). *PTPRQ* expression in the inner ear is confined to the hair bundles of the cochlea and vestibule, with its distribution varying by the type or location of the hair cells, primarily in the basal region of the hair bundles (Oziębło et al., 2019). In mice, *PTPRQ* is crucial for the formation of shaft connectors in the hair bundle, normal maturation of cochlear hair bundles, and the long-term survival of high-frequency auditory hair cells (Goodyear et al., 2003). *PTPRQ* ^−/−^ mice exhibit a reduction in stereocilia number in mutant hair bundles (Goodyear et al., 2012), and stereocilia in mice with *PTPRQ* variants typically develop during embryogenesis but deteriorate postnatally (Salles et al., 2014), leading to a loss of high-frequency auditory hair cells and deafness (Goodyear et al., 2012).

*PTPRQ* mutant mice exhibit malformations in shaft connectors and immature cochlear hair bundles, leading to deafness (Goodyear et al., 2003; Wu et al., 2018). However, the pathogenesis in humans remains unclear. Most cases of ARNSHL are attributed to hair cell dysfunction. The hair bundle atop the hair cell comprises approximately 100 static cilia laden with actin, facilitating the conversion of mechanical signals into electrical ones (Wang and Zhou, 2021). Additionally, inositol phospholipids are pivotal regulators of the actin cytoskeleton and membrane trafficking (Takenawa and Itoh, 2001). As a membrane-associated inositol lipid phosphatase, *PTPRQ* may regulate the local phosphoinositide content of the hair cells’ apical membrane (Goodyear et al., 2003; Oganesian et al., 2003). Using quantitative PCR analysis on fragments encoding the intracellular region of *PTPRQ*, Schraders et al. demonstrated its expression in all but two tested human fetal tissues, with the highest expression in the fetal kidney, followed by the lung and cochlea (Schraders et al., 2010). Further studies have shown that *PTPRQ* possesses both phosphotyrosine phosphatase and phosphatidylinositol phosphatase activities, influencing cell proliferation, apoptosis, differentiation, and survival (Zhang et al., 2022).

We hypothesize that *PTPRQ* variants result in the synthesis of truncated proteins that cannot sustain normal physiological functions, thereby leading to organismal dysfunction. Nevertheless, the precise mechanisms underlying our findings require further validation.

As the identification of genes associated with deafness expands, efforts to develop viable and effective treatments for inherited hearing loss have intensified, alongside an emphasis on proactive prevention. Traditional interventions such as hearing aids and cochlear implants have limitations: cochlear implants are expensive and produce inconsistent outcomes, whereas hearing aids require a degree of residual hearing, thus restricting their utility. Consequently, there is increasing interest in innovative treatments like gene therapy, which aims to reduce hearing loss and restore cochlear function. Recent studies have successfully established various mouse models, strengthening the foundation for gene therapy research. However, considerable debate persists regarding methods of gene delivery and the choice of vectors. Due to the limitations imposed by the size of a single AAV vector, Lv et al., 2024 administered the AAV1-hOTOF dual vector via round-window injections to six patients suffering from severe to profound autosomal recessive deafness. All but one patient exhibited significant hearing recovery (Lv et al., 2024). Initial signs of hearing improvement were noted 4–6 weeks after injection in responsive patients, with ongoing improvement over time, illustrating a time-dependent recovery of hearing (Lv et al., 2024). This marks the first documented case of hearing restoration in congenitally deaf patients via gene therapy (Reisinger and Trapani, 2024). Following this, a dose of 1.5 × 10^12^ vg of the AAV1-hOTOF dual vector was administered to the bilateral inner ears of five children by the team, leading to bilateral hearing improvement, enhanced auditory and speech capabilities, and restored sound source localization (Wang et al., 2024). These outcomes demonstrate the feasibility of binaural gene therapy for DFNB9 patients using the AAV1-hOTOF dual vector, offering a novel method for the clinical translation of gene therapy for genetic deafness caused by other genes (Wang et al., 2024). Additionally, the co-regulation and interactions among proteins are crucial in the early development of cochlear morphology, affecting the timing of gene therapy (Liu and Rask-Andersen, 2022), the age window for gene therapy intervention remains to be determined through further research. Despite progress in gene therapy for hearing loss, research on *PTPRQ* and its involvement in auditory impairment is limited, highlighting the need for comprehensive functional studies on *PTPRQ* variants. Future research could explore personalized gene therapy targeting specific variants.

In conclusion, our study has identified two novel variants in the *PTPRQ* gene associated with deafness. These variants lead to the production of truncated *PTPRQ* proteins, which are likely contributors to hearing loss. Further investigations will be conducted on these variants to elucidate the underlying pathogenic mechanisms. Our findings suggest an association between the compound heterozygous variant of *PTPRQ* and the observed hearing phenotypes. However, the absence of ethnolinguistic hearing controls from the same ethnic group limits our study. Including such controls would help confirm the specificity of the association between these variants and the hearing phenotypes. In future research, we aim to determine the frequency of these variants in unaffected individuals from identical ethnic and residential backgrounds to further validate their role in hearing phenotypes. Additionally, while most existing studies have utilized mutant mice as animal models, indicating significant vestibular dysfunction alongside hearing loss, few reported cases have explicitly confirmed vestibular dysfunction (Goodyear et al., 2012; Sakuma et al., 2015). This discrepancy may stem from developmental differences in the cochlea between rodents and humans, necessitating further investigation into *PTPRQ*’s role in hearing loss.

## Data Availability

The raw data supporting the conclusions of this article will be made available by the authors, without undue reservation.
